# Supplementary material for: The Pathological and Histopathological Findings in Cats with Clinically Recognised Hypertrophic Cardiomyopathy Are Related to the Severity of Clinical Signs and Disease Duration
Source: Animals (Basel). 2025 Feb 27;15(5):703. doi: 10.3390/ani15050703 (PMC11898935; doi:10.3390/ani15050703)
Supplement: Supplementary file 1 [file animals-15-00703-s001.zip › animals-3476636-supplementary/suppl/Supplementary File 1.pdf]

Supplementary File S1 – the criteria serving for clinical diagnosis of hypertrophic cardiomyopathy

The criteria serving for the clinical diagnosis of hypertrophic cardiomyopathy (HCM)

- diffuse or regional increased left ventricular wall thickness with a nondilated left ventricular chamber: interventricular septum and/or left ventricular posterior wall thickness >6 mm[1]
  - measurements of the left ventricular wall thickness in 2D-guided M-mode echocardiographic measurements (both in left parasternal short-axis view and left parasternal long-axis view) supplemented with 2D echocardiographic measurements (in similar views) in cases of focal hypertrophy[1]
  - the measurements performed using leading edge-to-leading edge technique[1]
- exclusion of systemic hypertension, hyperthyroidism, diabetes mellitus, chronic kidney disease, and neoplastic disease (cardiac or extracardiac) based on clinical history, blood pressure measurements and blood examination[1]
- exclusion of long-term steroid administration based on clinical history

[1] Luis Fuentes, V., Abbott, J., Chetboul, V., Côté, E., Fox, P.R., Häggström, J. et al., 2020. ACVIM consensus statement guidelines for the classification, diagnosis, and management of cardiomyopathies in cats. Journal of Veterinary Internal Medicine 34, 1062–1077.
