# Supplementary material for: The Pathological and Histopathological Findings in Cats with Clinically Recognised Hypertrophic Cardiomyopathy Are Related to the Severity of Clinical Signs and Disease Duration
Source: Animals (Basel). 2025 Feb 27;15(5):703. doi: 10.3390/ani15050703 (PMC11898935; doi:10.3390/ani15050703)
Supplement: Supplementary file 1 [file animals-15-00703-s001.zip › animals-3476636-supplementary/suppl/Supplementary Table S1.pdf]

Supplementary Table S1. The clinical signs and survival time in cats diagnosed with hypertrophic cardiomyopathy included in the study.

Case number	Auscultation disorders	Dyspnoea	Pulmonary oedema	Pleural effusion	Pericardial effusion	ATE	IVSd [mm]	LVPWd [mm]	LA/Ao	Duration of treatment
1	+ muffled heart sounds	+	-	-	+	-	16.4	11.5	enlarged	< 1 week
2	+ heart murmur and gallop rhythm	-	-	+	-	-	6.0	5.7	3.3	7 months
3	-	-	-	+	-	+	Concentric hypertrophy		enlarged	<1 week
4	-	+	-	-	-	+	Concentric hypertrophy		enlarged	<1 week
5	-	-	-	-	-	+	Concentric hypertrophy		enlarged	<1 week*
6	-	+	-	-	-	-	Concentric hypertrophy		enlarged	2 years
7	-	+	-	+	+	+	Concentric hypertrophy		5.5	6 months
8	+ muffled heart sounds	-	-	-	+	+	Concentric hypertrophy		enlarged	<1 week
9	+ heart murmur	-	-	+	-	-	Concentric hypertrophy		enlarged	<1 week
10	-	+	+	-	-	-	Concentric hypertrophy		enlarged	<1 week*
11	-	-	+	-	-	+	Concentric hypertrophy		enlarged	<1 week
12	-	+	-	+	-	-	Concentric hypertrophy		enlarged	<1 week
13	-	+	-	+	-	-	Concentric hypertrophy		enlarged	<1 week
14	-	-	+	-	-	+	Concentric hypertrophy		enlarged	<1 week
15	-	+	+	-	-	+ (3 times)	7.1	11.1	2.31	2 years
16	-	-	-	+	-	+	Concentric hypertrophy		enlarged	<1 week
17	-	+	-	-	-	+	Concentric hypertrophy		enlarged	<1 week

18	-	+	-	-	-	-	Concentric hypertrophy		enlarged	<1 week*
19	-	+	-	+	-	-	5.9	6.9	2.86	3 years
20	-	+	+	-	-	-	Concentric hypertrophy		3.94	<1 week*
21	+ tachycardia	+	+	-	-	-	Concentric hypertrophy		3.5	8 years
22	+ gallop rhythm	-	-	+	-	-	Concentric hypertrophy		enlarged	<1 week
23	-	+	-	+	-	-	Concentric hypertrophy		2.0	<1 week
24	-	-	-	-	-	+	Concentric hypertrophy		enlarged	<1 week
25	-	+	+	-	-	-	6.0	7.9	2	2 years
26	-	+	-	-	-	+	6.0	6.5	2.47	<1 week
27	-	+	-	-	+	-	7.1	8.7	3.19	2 years
28	+ tachycardia	+	-	+	+	-	7.1	4.0	3.22	1 year
29	+ muffled heart sounds	+	+	-	-	+	Concentric hypertrophy		enlarged	<1 week
30	+ muffled heart sounds, heart murmur, arrhythmia	+	-	+	-	+	Concentric hypertrophy		2.45	<1 week
31	+ heart murmur	-	-	-	-	-	7.5	5.5	1.07	1 year*
32	+ heart murmur, muffled heart sounds, gallop rythm	+	+	+	+	-	6.2	7.5	2.8	4 months*
33	-	+	+	-	-	+	Concentric hypertrophy		enlarged	<1 week
34	+ heart murmur, tachycardia	+	+	-	-	-	5.3	7.2	1.36	4 months

ATE – aortic thromboembolism; IVSd – interventricular septum diameter in diastole; LVPWd – left ventricular posterior wall diameter in diastole; LA/Ao – left atrium-to-aorta ratio; + – symptom present (score 1); - – symptom absent (score 0); * patients presenting sudden death
