# Supplementary material for: The Pathological and Histopathological Findings in Cats with Clinically Recognised Hypertrophic Cardiomyopathy Are Related to the Severity of Clinical Signs and Disease Duration
Source: Animals (Basel). 2025 Feb 27;15(5):703. doi: 10.3390/ani15050703 (PMC11898935; doi:10.3390/ani15050703)
Supplement: Supplementary file 1 [file animals-15-00703-s001.zip › animals-3476636-supplementary/suppl/Supplementary Table S2.pdf]

Supplementary Table S2. The results of post-mortem gross examination of cats enrolled in the study.

Case number	Heart height [mm]	Heart width [mm]	LAA height [mm]	LAA width [mm]	IVSt	LVPWt
1	45.7	35.6	14.0	24.0	11.2	8.8
2	57.0	43.5	33.8	28.4	9.1	10.3
3	58.0	40.3	20.5	18.7	6.4	13.5
4	47.0	28.1	24.3	12.8	8.5	8.5
5	54.0	32.9	20.1	16.3	9.0	7.7
6	44.7	29.6	13.5	17.4	8.8	9.9
7	63.5	46.0	21.7	23.6	9.2	13.0
8	59.6	36.4	36.4	25.2	7.4	12.0
9	33	31	n/a	n/a	7.5	8.6
10	33.3	30.1	15.0	19.6	9.6	10.0
11	50.2	33.3	16.9	14.5	7.2	9.6
12	51.2	42.3	30.5	20.6	10.8	9.7

13	39.2	28.8	11.8	13.1	11.3	7.9
14	43.3	30.6	20.9	18.5	10.5	9.6
15	61.7	38.8	38.0	34.2	9.1	10.9
16	43.4	33.8	29.4	22.1	8.1	11.3
17	41.9	29.2	15.9	16.3	8.9	8.3
18	41.0	31.4	18.1	16.4	9.5	12.1
19	58.5	34.7	29.0	25.3	10.2	12.4
20	54.7	35.0	27.8	21.8	9.01	10.6
21	48.3	34.9	21.2	17.4	5.8	6.9

22	48.0	34.5	19.5	17.2	6.3	7.9
23	57.7	45.5	34.3	26.5	9.2	7.7
24	40.3	36.2	23.8	19.9	7.7	8.8
25	59.0	34.6	26.6	26.3	9.6	11.6
26	46.0	33.4	22.7	20.6	7.7	9.6
27	44.2	32.7	19.7	25.7	7.9	8.9
28	51.8	37.9	27.7	19.6	6.5	5.7
29	34.2	25.4	15.3	12.6	9.1	7.8
30	49.3	42.3	18.3	18.2	6.0	7.9
31	56.5	38.9	22.1	24.7	11.6	12.2
32	39.3	29.0	16.9	19.6	9.4	8.9
33	41.7	38.0	24.6	20.8	7.2	11.0

34	54.1	38.9	27.0	23.5	10.4	8.5

LAA – left atrial appendage; IVSt – interventricular septum thickness; LVPWt – left ventricular posterior wall thickness.
