# Supplementary material for: The Pathological and Histopathological Findings in Cats with Clinically Recognised Hypertrophic Cardiomyopathy Are Related to the Severity of Clinical Signs and Disease Duration
Source: Animals (Basel). 2025 Feb 27;15(5):703. doi: 10.3390/ani15050703 (PMC11898935; doi:10.3390/ani15050703)
Supplement: Supplementary file 1 [file animals-15-00703-s001.zip › animals-3476636-supplementary/suppl/Supplementary Table S3.pdf]

Supplementary Table S3. The results histopathologic examination of cats enrolled in the study

Case number	LV	IVS	RV	LA	RA
1	cardiomyocyte degeneration (++) and hypertrophy (+); myocardial fibrosis (+); fatty replacement (+); inflammatory infiltration (++; LYM) also in endocardium	cardiomyocyte degeneration (++) and hypertrophy (+); myocardial fibrosis (+); fatty replacement (+); inflammatory infiltration (++; LYM) also in endocardium	fatty replacement (++) with myocardial fibrosis (++); foci of inflammatory infiltration (LYM)	cardiomyocyte degeneration and lysis (++); fatty replacement (+++); myocardial fibrosis (+); inflammatory infiltration (++) also in the endocardium	myocardial degeneration (+++); fatty replacement (++)
2	cardiomyocyte degeneration (++); myocardial fibrosis (+++) with endocardial thickening and fibrosis (+++); single foci of inflammatory infiltration (+; LYM);	cardiomyocyte degeneration (++); myocardial fibrosis (+++) with endocardial thickening and fibrosis (+++); single foci of inflammatory infiltration (+; LYM);	cardiomyocyte degeneration (+); myocardial fibrosis (+)	cardiomyocyte degeneration (++); myocardial fibrosis (++); inflammatory infiltration (+; LYM)	cardiomyocyte degeneration (+); myocardial fibrosis (+)
3	cardiomyocyte degeneration (+); myocardial fibrosis (++)	cardiomyocyte degeneration (+); myocardial fibrosis (++)	cardiomyocyte degeneration (+); myocardial fibrosis (+++)	cardiomyocyte degeneration (++); myocardial fibrosis (+)	cardiomyocyte degeneration (++); myocardial fibrosis (+++)
4	cardiomyocyte degeneration (+++) and hypertrophy (++); areas of disarray	cardiomyocyte degeneration (+++) and hypertrophy (++); areas of disarray	cardiomyocyte degeneration (++)	cardiomyocyte degeneration (++)	cardiomyocyte degeneration (++)
5	cardiomyocyte degeneration (+) and hypertrophy (++); areas of disarray	cardiomyocyte degeneration (+) and hypertrophy (++); areas of disarray	normal	myocardial fibrosis (+)	cardiomyocyte degeneration (+); myocardial fibrosis (+)
6	cardiomyocyte degeneration (+++) and hypertrophy (+++); areas of disarray	cardiomyocyte degeneration (+++) and hypertrophy (+++); areas of disarray	cardiomyocyte degeneration (+++) and hypertrophy (+++)	endocardial thickening	myocardial fibrosis (+)
7	cardiomyocyte degeneration (++) and hypertrophy (++); areas of disarray	cardiomyocyte degeneration (++) and hypertrophy (++)	cardiomyocyte degeneration (++) and hypertrophy (++)	cardiomyocyte degeneration (++) and hypertrophy (+)	cardiomyocyte degeneration (+)

8	cardiomyocyte degeneration (+++) and hypertrophy (++); areas of disarray	cardiomyocyte degeneration (+++) and hypertrophy (++); areas of disarray	cardiomyocyte degeneration (+++)	cardiomyocyte degeneration (++)	cardiomyocyte degeneration (++)
9	single area of myocardial lysis	normal	focal fatty replacement (++)	normal	fatty replacement (++); myocardial lysis with replacement fibrosis (++)
10	cardiomyocyte degeneration (+); fatty replacement (++)	normal	cardiomyocyte degeneration (+); fatty replacement (+++)	cardiomyocyte degeneration (+); fatty replacement (+)	cardiomyocyte degeneration (+); fatty replacement (+++)
11	cardiomyocyte degeneration (++) with hypertrophy (+); myocardial fibrosis (++); foci of inflammatory infiltration (+; LYM)	cardiomyocyte degeneration (++) with hypertrophy (+); myocardial fibrosis (++); foci of inflammatory infiltration (+; LYM)	cardiomyocyte degeneration (+)	cardiomyocyte degeneration (+)	cardiomyocyte degeneration (+)
12	cardiomyocyte degeneration (++)	cardiomyocyte degeneration (++)	cardiomyocyte degeneration (++)	cardiomyocyte degeneration (++); myocardial fibrosis (+++); inflammatory infiltration (LYM-PLA; ++)	cardiomyocyte degeneration (++); myocardial fibrosis (++); inflammatory infiltration (LYM-PLA; ++)
13	cardiomyocyte degeneration (+++)	cardiomyocyte degeneration (+++)	cardiomyocyte degeneration (+); fatty replacement of myocardium (++)	fatty replacement of myocardium (+); single inflammatory cells under epicardium; cardiomyocyte degeneration (+)	cardiomyocyte degeneration (+)
14	cardiomyocyte degeneration (++) and hypertrophy (+++); areas of cardiomyocyte necrosis (+++); areas of mixed inflammatory infiltration (NEU-LYM; +++)	cardiomyocyte degeneration (++) and hypertrophy (+++); areas of cardiomyocyte necrosis (+++); areas of mixed inflammatory infiltration (NEU-LYM; +++)	cardiomyocyte degeneration (+); inflammatory infiltration (NEU-LYM; ++)	cardiomyocyte degeneration (+); inflammatory infiltration (NEU-LYM; +)	cardiomyocyte degeneration (+++)

15	cardiomyocyte degeneration (+++) and hypertrophy (+++); areas of myocardial necrosis; areas of myocardial disarray	cardiomyocyte degeneration (+++) and hypertrophy (+++); areas of myocardial necrosis; areas of myocardial disarray	cardiomyocyte degeneration (+)	cardiomyocyte degeneration (+++); myocardial necrosis (+); endocardial thickening	cardiomyocyte degeneration (+)
16	cardiomyocyte degeneration (++) and hypertrophy (++); foci of disarray; small foci of myocardial necrosis;	cardiomyocyte degeneration (++) and hypertrophy (++); foci of disarray; small foci of myocardial necrosis;	cardiomyocyte degeneration (+)	cardiomyocyte degeneration (+++); foci of necrosis; foci of inflammatory infiltration (+; LYM); endocardial thickening	cardiomyocyte degeneration (+)
17	cardiomyocyte degeneration (+++) and necrosis (++); inflammatory infiltration (NEU-LYM; +)	cardiomyocyte degeneration (+++) and necrosis (++); inflammatory infiltration (NEU-LYM; +)	cardiomyocyte degeneration (+); fatty replacement (+); perivascular inflammatory infiltration (NEU-LYM; +)	cardiomyocyte degeneration (++); inflammatory infiltration in the myocardium and endocardium (NEU-LYM; +++)	cardiomyocyte degeneration (++); myocardial fibrosis (+); fatty replacement (++)
18	cardiomyocyte degeneration with the presence of multiple vacuole (+++); myocardial fibrosis (++)	cardiomyocyte degeneration with the presence of multiple vacuole (+++); myocardial fibrosis (++)	cardiomyocyte degeneration with the presence of multiple vacuole (+++); myocardial fibrosis (++)	myocardial fibrosis (+)	myocardial fibrosis (++)
19	cardiomyocyte degeneration (+++); myocardial fibrosis (+++)	cardiomyocyte degeneration (+++); myocardial fibrosis (+++)	cardiomyocyte degeneration (+++); myocardial fibrosis (++)	cardiomyocyte degeneration (+++)	cardiomyocyte degeneration (+++)
20	cardiomyocyte degeneration (++); myocardial fibrosis (++); multiple areas of disarray	cardiomyocyte degeneration (++); myocardial fibrosis (++); multiple areas of disarray	cardiomyocyte degeneration (+)	cardiomyocyte degeneration (+); multiple inflammatory infiltrates in the epicardium (+++; LYM)	cardiomyocyte degeneration (+); multiple inflammatory infiltrates in the epicardium (+; LYM)
21	cardiomyocyte degeneration (++); myocardial fibrosis (+++)	cardiomyocyte degeneration (++); myocardial fibrosis (+++)	cardiomyocyte degeneration (++)	myocardial fibrosis (+++); single foci of inflammatory infiltration (LYM)	cardiomyocyte degeneration (++)

22	cardiomyocyte degeneration (+); myocardial fibrosis (+); areas of disarray	cardiomyocyte degeneration (+)	cardiomyocyte degeneration (+)	cardiomyocyte degeneration (+)	cardiomyocyte degeneration (+)
23	cardiomyocyte degeneration (+++) and hypertrophy (+++); myocardial fibrosis (+)	cardiomyocyte degeneration (++)	cardiomyocyte degeneration (++)	cardiomyocyte degeneration (+++); single foci of inflammatory infiltration (LYM)	cardiomyocyte degeneration (+)
24	cardiomyocyte degeneration (+) and hypertrophy (+); areas of myocardial disarray; myocardial fibrosis (+)	cardiomyocyte degeneration (+) and hypertrophy (+); areas of myocardial disarray; myocardial fibrosis (+)	cardiomyocyte degeneration (+)	cardiomyocyte degeneration (+)	cardiomyocyte degeneration (+)
25	cardiomyocyte degeneration (+++) and hypertrophy (+++); areas of disarray	cardiomyocyte degeneration (+++) and hypertrophy (+++); areas of disarray	cardiomyocyte degeneration (+++) and hypertrophy (+++)	cardiomyocyte degeneration (+++) and hypertrophy (+++)	cardiomyocyte degeneration (+++) and hypertrophy (+++)
26	cardiomyocyte degeneration (+) and hypertrophy (+); myocardial fibrosis (+) and lysis (++); inflammatory infiltration (LYM; +)	cardiomyocyte degeneration (+) and hypertrophy (+); myocardial fibrosis (+) and lysis (++); inflammatory infiltration (LYM; +)	cardiomyocyte degeneration (+) and hypertrophy (+); myocardial fibrosis (++); inflammatory infiltration (LYM; ++)	cardiomyocyte degeneration (+++); myocardial fibrosis (++); inflammatory infiltration (LYM: +++)	cardiomyocyte degeneration (+++)
27	cardiomyocyte degeneration (++); myocardial fibrosis (++); inflammatory infiltration in the epicardium (LYM; +++)	cardiomyocyte degeneration (++); myocardial fibrosis (++); inflammatory infiltration in the epicardium (LYM; +++)	cardiomyocyte degeneration (++)	cardiomyocyte degeneration (+++); myocardial fibrosis (+); inflammatory infiltration (LYM; ++)	cardiomyocyte degeneration (++); inflammatory infiltration (LYM; +)
28	cardiomyocyte degeneration (+++); myocardial fibrosis (++); thickening and fibrosis of endocardium (+++); inflammatory infiltration in epicardium (LYM; ++)	cardiomyocyte degeneration (+++); myocardial fibrosis (++); thickening and fibrosis of endocardium (+++); inflammatory infiltration in epicardium (LYM; ++)	cardiomyocyte degeneration (+++); myocardial fibrosis (+)	cardiomyocyte degeneration (+++); inflammatory infiltration in the epicardium (LYM; ++)	cardiomyocyte degeneration (++)

29	cardiomyocyte degeneration (+++); focal disarray	cardiomyocyte degeneration (+++); focal disarray	cardiomyocyte degeneration (+++)	cardiomyocyte degeneration (++); endocardial thickening	cardiomyocyte degeneration (+++)
30	cardiomyocyte degeneration (+); myocardial fibrosis (+); myocardial lysis (++); focal inflammatory infiltration (LYM; +)	cardiomyocyte degeneration (+); myocardial fibrosis (+); myocardial lysis (++); focal inflammatory infiltration (LYM; +)	cardiomyocyte degeneration (+); myocardial lysis (+); focal inflammatory infiltration (LYM; +)	cardiomyocyte degeneration (+); myocardial fibrosis (+++); inflammatory infiltration (LYM; +)	cardiomyocyte degeneration (+); myocardial fibrosis (++)
31	cardiomyocyte degeneration (++); cardiomyocyte hypertrophy (+); areas of disarray; myocardial fibrosis (+++); endocardial thickening (+)	cardiomyocyte degeneration (+); myocardial fibrosis (++)	cardiomyocyte degeneration (+); myocardial fibrosis (++)	cardiomyocyte degeneration (+); myocardial fibrosis (+); endocardial thickening (+)	cardiomyocyte degeneration (++); myocardial fibrosis (+)
32	cardiomyocyte degeneration (++) and hypertrophy (+++); myocardial fibrosis (++); endocardial thickening and fibrosis (++); focal inflammatory infiltration (LYM; +)	cardiomyocyte degeneration (++) and hypertrophy (+++); myocardial fibrosis (++); endocardial thickening and fibrosis (++); focal inflammatory infiltration (LYM; +)	cardiomyocyte degeneration (++) and hypertrophy (+++); myocardial fibrosis (++); focal inflammatory infiltration (LYM; +)	cardiomyocyte degeneration (+++) and hypertrophy (+++); myocardial fibrosis (+++); inflammatory infiltration (LYM; +++)	cardiomyocyte degeneration (++); myocardial fibrosis (+); inflammatory infiltration (LYM: +)
33	cardiomyocyte degeneration (+); myocardial fibrosis (+); inflammatory infiltration (LYM; ++)	cardiomyocyte degeneration (+); myocardial fibrosis (+); inflammatory infiltration (LYM; ++)	cardiomyocyte degeneration (+)	cardiomyocyte degeneration (++); myocardial fibrosis (+)	cardiomyocyte degeneration (+)
34	cardiomyocyte degeneration (+++) and hypertrophy (+); myocardial fibrosis (++); myocardial necrosis (+); inflammatory infiltration (NEU-LYM-PLA; +++)	cardiomyocyte degeneration (+++) and hypertrophy (+); myocardial fibrosis (++); myocardial necrosis (+); inflammatory infiltration (NEU-LYM-PLA; +++)	cardiomyocyte degeneration (+++); myocardial fibrosis (+); myocardial necrosis (++); inflammatory infiltration (NEU-LYM-PLA; +++); endocardial thickening with inflammatory infiltration (+++)	cardiomyocyte hypertrophy (+); myocardial fibrosis (++)	normal

LV – left ventricle; IVS – interventricular septum; RV – right ventricle; LA – left atrium; RA – right atrium LYM – lymphocytic; NEU – neutrophilic; PLA – plasmacytic; the severity of changes assessed in the 3-grade scale: (+) mild (score 1), (++) moderate (score 2), (+++) severe (score 3).
